# Dr. Jullius of Prussia

**Published:** 1835

**Authors:** 


### V.—Dr. Julius, of Prussia.

We have lately derived much gratification from several days' intercourse with this enlightened physician and civilian, sent to the United States by the king of Prussia, to inquire into the state of its institutions, especially its penetentiaries and other prisons. Dr. Julius is one of the editors of a medical periodical entitled "The Journal of Foreign Medicine," published in Hamburg, which we shali hereafter receive in exchange for this journal, and from which our learned friend, Professor Gross, will make such translations as may be interesting to our western readers. Dr. Julius is a gentleman of great observation, and having directed his attention to the state of the medical profession in the United States, will, it may be expected, on his return, furnish us with some sound critical advice, on our plans of study and systems of medical police. In a free conversation with him on the relative scope of studies in Prussia and the United States, we could not ward off a feeling of mortification, at the limited and superficial character of the latter compared with the former. We do not know that Dr. Juluis intends to write a book of travels, but we hope he will at least give us a memoir in his periodical, on the state of medical instruction and professional learning in America.

